# GmABCG5, an ATP-binding cassette G transporter gene, is involved in the iron deficiency response in soybean

**DOI:** 10.3389/fpls.2023.1289801

**Published:** 2024-01-05

**Authors:** Yu Wang, Xuemeng Zhang, Yuhan Yan, Tingting Niu, Miao Zhang, Chao Fan, Wenwei Liang, Yongjun Shu, Changhong Guo, Donglin Guo, Yingdong Bi

**Affiliations:** ^1^ Heilongjiang Provincial Key Laboratory of Molecular Cell Genetics and Genetic Breeding, College of Life Science and Technology, Harbin Normal University, Harbin, China; ^2^ Institute of Crops Tillage and Cultivation, Heilongjiang Academy of Agricultural Sciences, Harbin, China

**Keywords:** ATP-binding cassette, GmABCG5, iron deficiency, soybean, RNAi

## Abstract

Iron deficiency is a major nutritional problem causing iron deficiency chlorosis (IDC) and yield reduction in soybean, one of the most important crops. The ATP-binding cassette G subfamily plays a crucial role in substance transportation in plants. In this study, we cloned the *GmABCG5* gene from soybean and verified its role in Fe homeostasis. Analysis showed that GmABCG5 belongs to the ABCG subfamily and is subcellularly localized at the cell membrane. From high to low, *GmABCG5* expression was found in the stem, root, and leaf of young soybean seedlings, and the order of expression was flower, pod, seed stem, root, and leaf in mature soybean plants. The GUS assay and qRT-PCR results showed that the *GmABCG5* expression was significantly induced by iron deficiency in the leaf. We obtained the *GmABCG5* overexpressed and inhibitory expressed soybean hairy root complexes. Overexpression of *GmABCG5* promoted, and inhibition of *GmABCG5* retarded the growth of soybean hairy roots, independent of nutrient iron conditions, confirming the growth-promotion function of *GmABCG5*. Iron deficiency has a negative effect on the growth of soybean complexes, which was more obvious in the *GmABCG5* inhibition complexes. The chlorophyll content was increased in the *GmABCG5* overexpression complexes and decreased in the *GmABCG5* inhibition complexes. Iron deficiency treatment widened the gap in the chlorophyll contents. FCR activity was induced by iron deficiency and showed an extraordinary increase in the *GmABCG5* overexpression complexes, accompanied by the greatest Fe accumulation. Antioxidant capacity was enhanced when *GmABCG5* was overexpressed and reduced when *GmABCG5* was inhibited under iron deficiency. These results showed that the response mechanism to iron deficiency is more actively mobilized in *GmABCG5* overexpression seedlings. Our results indicated that *GmABCG5* could improve the plant’s tolerance to iron deficiency, suggesting that *GmABCG5* might have the function of Fe mobilization, redistribution, and/or secretion of Fe substances in plants. The findings provide new insights into the ABCG subfamily genes in the regulation of iron homeostasis in plants.

## 1 Introduction

Iron (Fe) is an indispensable trace element for plant growth. Iron often forms as insoluble Fe(III) oxyhydroxide in calcite soils, meaning that plants often suffer from iron deficiency (Ishimaru et al., 2006; Hindt and Guerinot, 2012). Because iron plays a crucial role in electron transport and chlorophyll formation, an adequate supply of iron in the chloroplast is required to perform the photosynthetic process. Iron deficiency alters photosynthesis and reduces chlorosis, or the yellowing of plant leaves (Pandey et al., 2021; Prity et al., 2021; Therby-Vale et al., 2022). Iron starvation is a major nutritional problem that causes severe visual symptoms and yield reductions in plants. The calcareous soils favor the development of iron deficiency chlorosis (IDC) in soybean (*Glycine max* L. Merr.) and result in soybean yield losses (Atencio et al., 2021; Merry et al., 2022).

Higher plants have developed effective strategies to enhance iron uptake in soils with low Fe availability, such as the reduction-based strategy (Strategy I) in nongraminaceous plants and the chelation-based strategy (Strategy II) in graminaceous species. When dicotyledonous plants are subjected to Fe-limited conditions, a proton pump, H^+^-ATPase2 (AHA2), contributes to the acidification of the rhizosphere to facilitate the solubilization of Fe(III); the ferric-chelate reductase reduces Fe(III) to Fe(II), and Fe(II) is then transported into root epidermal cells by Iron Regulated Transporter1 (IRT1) (Kobayashi et al., 2019). Plant iron transporters such as yellow stripe-like proteins (YSL), natural resistance-associated macrophage proteins (NRAMP), and vacuolar iron transporters (VIT) are involved in the iron uptake, sequestration, translocation, and remobilization processes (Kurt and Filiz, 2020). The ability to improve the availability and efficiency of Fe strongly affects both crop yield and quality. Understanding the mechanisms of Fe uptake, transport, and storage is essential for breeding crops that are more tolerant to Fe-limited conditions. The iron transporter genes can be used as genetic improvement target genes to increase plant tolerance to iron deficiency (Curie et al., 2001).

Multiple phytohormones, such as auxin, abscisic acid (ABA), ethylene, and brassinosteroids, regulate the Fe deficiency response (Briat et al., 2012). Previous studies have shown that ABA participates in Fe reutilization and transport under Fe-deficient conditions (Zhang et al., 2019). The involvement of auxin in the regulation of the Fe deficiency response is also well known. Recently, it was demonstrated that Arabidopsis roots enhance the elongation of cells leaving the root meristem under Fe deficiency depend on the auxin-mediated response, indicating that auxin acts downstream in the transmission of the Fe deficiency signal (Giehl et al., 2012; Wu et al., 2012; Lin et al., 2016). Furthermore, the Fe deficiency-caused alterations in *IRT1* and citrate synthase2 (*PbCS2*) expression are modulated by auxin (Blum et al., 2014; Li et al., 2016). These results strongly suggest that the iron deficiency-induced physiological responses are mediated by systemic auxin signaling.

ATPase-binding cassette (ABC) transporters are membrane transport proteins that use the energy released by the hydrolysis of ATP (Lefevre et al., 2018a). ABCs mediate the transport of hormones, pigments, toxic chemicals, secondary metabolites, lipid molecules, reactive oxygen species-related compounds, and heavy metal ions (Linton and Higgins, 2007; Kang et al., 2015; Hwang et al., 2016). In addition to transport, ABCs also act as ion channels and molecular switches to regulate a variety of cellular functions and physiological processes (Hinrichs et al., 2017; Zaynab et al., 2022). From microorganisms to mammals, members of the ATP-binding cassette G subfamily (ABCG subfamily) play a crucial role (Mcfarlane et al., 2010; Borghi et al., 2015; Dhara and Raichaudhuri, 2021; Niu et al., 2021). Genome studies have shown that the ABCG subfamily members are particularly abundant in plants, surpassing other eukaryotes such as human beings and yeast (Andolfo et al., 2015; Gupta et al., 2019). Transport substrates of ABCG transporters include terpenoids, flavonoids, plant hormones, and heavy metals in plants.

The ABCG subfamily includes two categories: White-brown Complex Homolog (WBC) and Pleiotropic Drug Resistance (PDR), both of which are involved in metal homeostasis. The full-length ABC transporter AtABCG37 (alias PDR9) mediates the secretion of the phenolic compound coumarin from Arabidopsis roots into the medium in response to iron deficiency. Expression of *AtABCG37* was up-regulated by iron deficiency in Arabidopsis roots (Fourcroy et al., 2014; Ziegler et al., 2017). The *Atabcg19* mutant had reduced iron uptake, accompanied by upregulation of the gene encoding iron reduction oxidase 6 (Mentewab et al., 2014). Expression of *NtABCG3* (*NtPDR3*) in the tobacco root epidermis was strongly induced by iron deficiency. Silencing and overexpression experiments showed that *NtABCG3* mediates the secretion of *O*-methylated coumarin into the rhizosphere in response to iron deficiency (Lefèvre et al., 2018b).

Transmembrane transport of ABA by ABCG transporters is one of the important methods in plants (Kuromori et al., 2010; Hwang et al., 2016; Kuromori et al., 2017; Pawela et al., 2019; Zhang et al., 2021; Kim et al., 2022). Hyperaccumulation of radioactive indole-3-butyric acid was observed in the root tips of *AtABCG36* (*atpdr8*) seedlings (Strader and Bartel, 2009). Analyses of single and double-mutant phenotypes suggest that both *ABCG36* and *ABCG37* function cooperatively in auxin-controlled plant development. The effect of *ABCG1* and *ABCG16* promoted rapid pollen tube growth through their effects on auxin distribution and auxin flow in the pistil (Liu et al., 2020). The ABC transporter AtPGP4 is involved in auxin-mediated lateral root and root hair development (Santelia et al., 2005). In the ABCB subfamily, *OsABCB14* was supported as an auxin transporter involved in Fe homeostasis (Xu et al., 2014).

Human ABCG5 functions in the intestinal absorption and biliary elimination of plant sterols and cholesterol, defects that result in sitosterolemia (Yu et al., 2005; Moitra and Dean, 2011; Lee et al., 2016). In Arabidopsis, ABCG5-mediated formation of a dense cuticle layer was required for early seedling establishment (Do et al., 2018). The *atabcg5* seedlings failed to develop true leaves when grown under waterlogged conditions (Lee et al., 2021). The *OsABCG5* from *Oryza sativa* is involved in the hypodermal suberization of roots and positively controls shoot branching by promoting the outgrowth of lateral shoots, known as *reduced culm number1* (*rcn1*) (Yasuno et al., 2009; Shiono et al., 2014). *OsABCG5* positively affects shoot branching independently of auxin signaling, while it is proposed to act on ABA translocation in guard cells (Yasuno et al., 2009; Matsuda et al., 2016). The orthologous of the *OsABCG5* gene, *LpABCG5*, was identified in perennial ryegrass as a candidate for the plant type determinant (Shinozuka et al., 2011). *NtABCG5* was reported to be involved in short- and long-term defense against insect/herbivore attacks by secreting toxic substrates (Bienert et al., 2012). The results indicate that *ABCG5* plays an important role in the growth and development of monocotyledons.

Soybean is one of the most important crops in the world and is sensitive to iron deficiency. Iron deficiency chlorosis (IDC) in calcareous soils seriously affects the economic and nutritional value of soybean (Waters et al., 2018; Merry et al., 2022). Among soybean ABC families, the ABCG subfamily has the largest number of GmABCs with 117, or 44.8% (Verrier et al., 2008; Mishra et al., 2019; Huang et al., 2021). At present, there are few studies on the function and identification of the *ABCG* genes in soybean plants. Nevertheless, the function of the *ABCG5* gene in dicotyledonous plants has been poorly investigated.

ABC transporter functions have been proposed using homologous expression and RNAi-silenced plant systems (Kim et al., 2007; Song et al., 2010). We have obtained several soybean iron deficiency response genes by screening the iron deficiency transcriptome database, and one of these genes, *GmABCG5* (LOC100789722), was chosen for further study (Li et al., 2023). In order to verify the role of *GmABCG5* in Fe homeostasis, we characterized the *GmABCG5* gene using homologous expression and RNAi-silenced plant systems. The results will clarify the function of the *GmABCG5* gene and provide new insights into the regulation of iron homeostasis in soybean.

## 2 Materials and methods

### 2.1 Plant materials and growth conditions

Suinong37 (SN37) and William82 soybeans were donated by the Institute of Crops Tillage and Cultivation, Heilongjiang Academy of Agricultural Sciences. SN37 seeds were hydroponically grown in 1/2 Hoagland, and roots, stems, and leaves at the three-leaf stage were sampled. the roots, stems, leaves, flowers, 1-2 cm young pods, and mature seeds of SN37 soybean planted in the experimental field were sampled. William82 soybean was used for physiological index detection and GUS histochemical staining. All plants were grown in a greenhouse at 16/8 h (light/dark, 22/20°).

### 2.2 Cloning and bioinformatic analysis of *GmABCG5* and *GmABCG5* promoter

Total RNA was extracted from SN37 soybean seedlings using Total RNA Kit II (Omega BioTek, Norcross, GA, USA). The cDNA was synthesized using ReverTra Ace™ qPCR RT Master MIX (TOYOBO, Japan). The coding sequence of *GmABCG5* was amplified from the cDNA. The total DNA of SN37 was extracted using the E.Z.N.A.® SP Plant DNA Kit (OMGAE, USA). After purification, the PCR products were sequenced (Sangon Biotechnology Co. Ltd., Shanghai, China) and analyzed (https://www.ncbi.nlm.nih.gov/orffinder/). From NCBI (https://www.ncbi.nlm.nih.gov) and Phytozome (https://phytozome-next.jgi.doe.gov/info/Gmax_Wm82_a2_v1) the *GmABCG5* protein sequence was *GmABCG5* promoter analysis by Plant CARE (http://bioinformatics.psb.ugent.be/webtools/plantcare/html/). The ABC protein sequence in the evolutionary tree was downloaded from NCBI and Phytozome and analyzed with MEGA7.0. Chromosome localization was analyzed with the TBtools software. Introns were analyzed with GSDS: http://GSDS.cbi.pku.edu.cn.

### 2.3 Expression analysis of *GmABCG5*

SN37 soybeans were grown in a 1/2 Hoagland nutrient solution to the three-leaf stage and then treated with iron deficiency for 7 d. The isolated RNA was used as a template, and qRT-PCR analysis was performed using SYBR®TremixExTaqTMII (TAKARA, Otsu, Japan) on CFX96 real-time PCR detection systems (Bio-Rad, Hercules, CA, USA). The *GmACTIN* gene was selected as an internal reference gene and was used as an internal control to normalize the data (Jian et al., 2008). The relative expression of genes was calculated by ▵▵Ct. The primers used are listed in **Supplementary Table 1**.

### 2.4 Subcellular localization of GmABCG5

The *GmABCG5* gene fragment without a stop codon was connected to GFP and expressed under the CaMV 35S promoter to construct the pBI121-GmABCG5-GFP recombinant vector. The 35S::*GmABCG5*-GFP fusion vector and the 35S::GFP vector (the control) were transformed into Agrobacterium GV3101 by the freeze-thaw method and then separately infiltrated into the leaves of a 3-week-old *Nicotiana benthamiana* leaf using 1-mL needless syringes. After 48 h, the GFP fluorescence was detected at 488 nm using a Zeiss LSM 510 Meta confocal laser scanning microscope with a 500-530 nm emission filter (Zeiss, Oberkochen, Germany).

### 2.5 GUS staining and determination of GUS enzyme activity

The pCAMBIA1301-*GmABCG5*pro::GUS recombinant vector was constructed and transformed into Agrobacterium K599 by the freeze-thaw method, and soybean hairy roots were induced by needle insertion. The soybean complex was transferred to 1/2 Hoagland nutrient solution and 1/2 Hoagland nutrient solution without iron supply for 7 d. Tissue staining was conducted following the method described previously (Li et al., 2023). The stained tissues were photographed using a stereomicroscope (EZ4-HD LEICA, Germany) coupled with a color charge-coupled device (CCD) camera (Zeiss, Germany). The GUS enzyme activity was detected using a plant β-glucuronidase (GUS) ELISA kit. The absorbance (OD value) was measured at 450nm wavelength, with the standard concentration as the horizontal coordinate and the corresponding OD value as the ordinate. The standard linear regression line was drawn, and the GUS enzyme activity value was calculated according to the linear regression equation.

### 2.6 Vector construction and genetic transformation

The pROKII-*GmABCG5* overexpressed recombinant vector and the pZH01-*GmABCG5* RNAi recombinant vector driven by the CaMV 35S promoter were constructed. Healthy soybean seeds with full particles were selected, disinfected with a 15% sodium hypochlorite solution for 5 min, and sown in sterile soil (nutrient soil: vermiculite = 1:1). After 5-7 d, soybean seedlings with good growth status were selected, and the surrounding tissues were punctured with the head of a syringe 2-3 mm below the cotyledon node. The recombinant plasmid pROKII empty vector, pROKII-*GmABCG5*, and pZH01-*GmABCG5* were transformed into Agrobacterium K599 by the freeze-thaw method, and single bacterial populations were screened and smeared on the wound site. After inoculation, it was cultured in a warm and humid environment, and hairy roots grew after 9-12 d. The obtained hairy root complex of soybean is called empty vector (Ev), overexpression *GmABCG5* soybean complex (OE*GmABCG5*), and inhibition *GmABCG5* soybean complex (i*GmABCG5*). When the hairy root grew to 2-3 cm, it was buried with sterilized vermiculite and then cultured for 20-30 d. Hairy roots were detected by qRT-PCR.

### 2.7 Physiological indices of overexpression and inhibition of the *GmABCG5* soybean complex

The soybean complex was transferred to 1/2 Hoagland nutrient solution and 1/2 Hoagland nutrient solution without iron supply for 7 d. The ferric chelate reductase assay was conducted as described previously (Yi and Guerinot, 1996). A Perls staining solution was mixed with 4% hydrochloric acid [v/v] and 4% [w/v] potassium ferricyanide in equal proportions (Roschzttardtz et al., 2009). The content of chlorophyll was determined by the 80% acetone and the light absorption values of the solution was tested at 663 nm and 645 nm (Manzoor et al., 2022). The content of superoxide anion (O_2_^-^) and malondialdehyde (MDA) was determined according to Dhindsa and other experimental methods with some modifications (Rajinder et al., 1982). Peroxidase (POD) and catalase (CAT) activities in plants were analyzed according to the published protocol with minor modifications (Chen and Zhang, 2016). Plant parts were blotted dry, and the fresh weight (g) of leaves and roots was measured using a digital scale. Root length (cm) was measured using a ruler.

### 2.8 Statistical analysis

For each experiment, at least three plants were sampled. Statistical analyses were performed using the SPSS software, version 18.0. Data are shown as mean ± SD. Differences in means between two groups were compared using a two-tailed Student’s t-test and among three or more groups by one-way ANOVA followed by Duncan’s multiple-range test. Data analysis is shown in **Supplementary Tables 4-6**.

## 3 Results

### 3.1 Evolutionary analysis, structure, and chromosomal localization of *GmABCG5*

**Figure 1:**
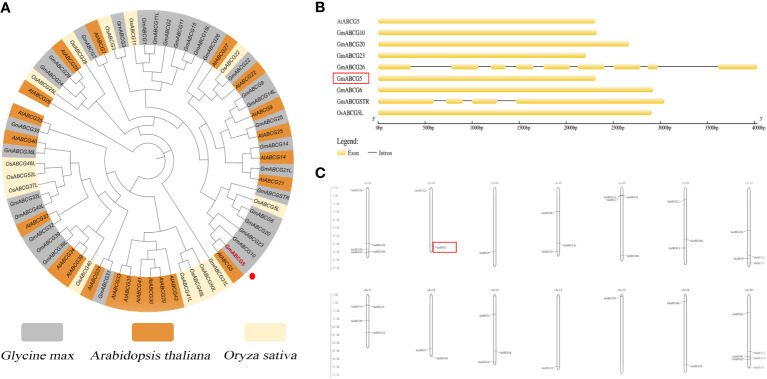
Bioinformatic analysis of *GmABCG5*. **(A)** Phylogenetic tree of the ABCG subfamily in *Arabidopsis thaliana*, *Glycine max*, and *Oryza sativa*. A neighbor-joining tree showed the evolutionary relationships of the ABCG members of the three species, which are highlighted in different colors. **(B)** Structure of some ABCG genes in *Arabidopsis thaliana*, *Glycine max*, and *Oryza sativa*. **(C)** Chromosomal mapping of 40 *GmABCG* genes in soybean. The scale on the left represents the chromosome length in megabases (Mb). The chromosome number is indicated to the left of each chromosome.

As shown in the ABCG subfamily phylogenetic tree, eight ABCGs (GmABCG23, GmABCGSTR, GmABCG10, GmABCG6, GmABCG20, AtABCG5, and OsABCG5) were assigned to the same branch. Soybean GmABCG5 was closely related to the AtABCG5 of *Arabidopsis thaliana* and the GmABCG10 of *Glycine max* (**Figure 1A**; **Supplementary Table 2**). There were no introns in *GmABCG5*, *AtABCG5*, and *OsABCG5* (**Figure 1B**). The 40 identified soybean *GmABCG* genes were unevenly distributed among the 14 chromosomes. The majority of the *GmABCG* genes weree located at the ends of the chromosomes. Some soybean chromosomes contained multiple *GmABCG* genes, for example, Ghr20 carried six *GmABCG* genes. *GmABCG5* was located at the end of soybean Ghr5, with no other *GmABCG* genes nearby (**Figure 1C**; **Supplementary Table 3**).

### 3.2 Expression of *GmABCG5*

**Figure 2:**
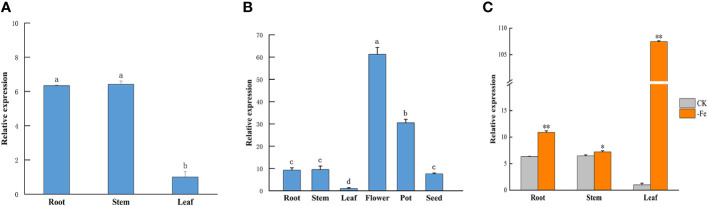
Tissue expression of *GmABCG5*. **(A)** Relative expression of *GmABCG5* in vegetative organs of Suinong37 soybean young seedlings. The roots, stems, and leaves of three-leaf stage soybean in hydroponic soybean were detected. **(B)** Relative expression of *GmABCG5* in the reproductive organs of Suinong37 soybean. The roots, stems and leaves, full-flowering flowers, 1-2 cm young pods, and mature seeds of field-grown Suinong37 soybean plants were detected. **(C)** *GmABCG5* gene expression in hydroponically grown soybean under iron deficiency for 7 d. Experiments were repeated three times, and each treatment group contained five samples. Data represent the mean of the replicates. Lowercase letters indicate *p*<0.05.

The qRT-PCR results showed that the *GmABCG5* expression ranged from high to low in the stem, root, and leaf of young soybean seedlings. There was not much difference in the expression of *GmABCG5* in stem and root, while the expression of *GmABCG5* in leaves was significantly lower than that in stem and root (*p*<0.05) (**Figure 2A**). The expression level of *GmABCG5* from high to low was flower, pod, seed, root, stem, and leaf in mature soybean plants. The *GmABCG5* expression was significantly higher in the flower than that in the pod and seed, and was significantly higher in the pod than that in the seed (*p*<0.05) (**Figure 2B**).

### 3.3 GmABCG5 localized at the cell plasma membrane

**Figure 3:**
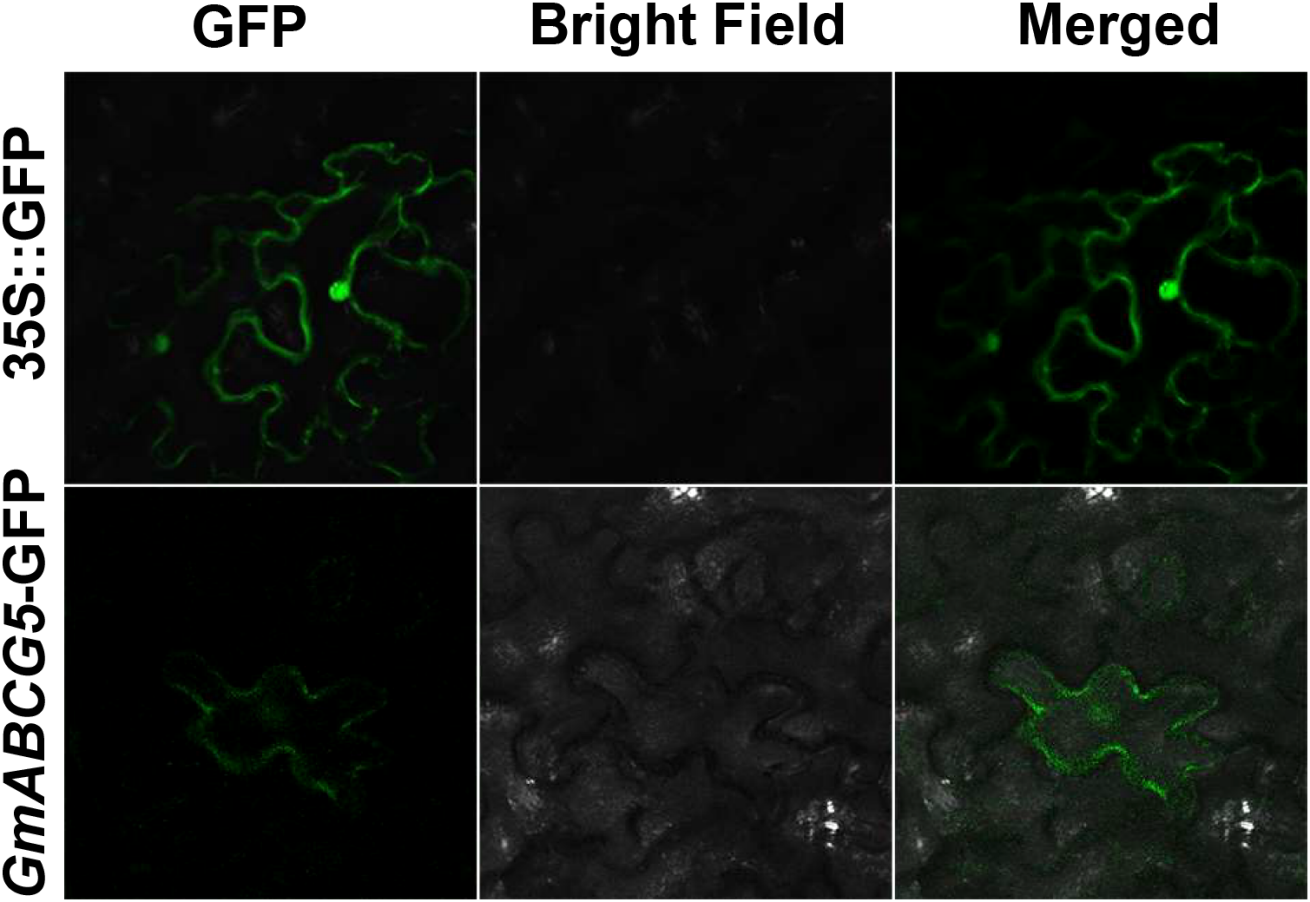
Subcellular localization of GmABCG5. The fusion protein 35S::*GmABCG5*-GFP was transiently transformed into epidermal cells of *Nicotiana benthamiana* leaves. 35S::GFP was used for comparison. These images were obtained using a confocal microscope. Bar = 100 µm.

GmABCG5 was fused to the green fluorescent protein (GFP) and driven by a 35S promoter. The 35S::*GmABCG5*-GFP fluorescence signal was observed exclusively at the plasma membrane of the epidermal cells of the *Nicotiana benthamiana* leaves, while the 35S::GFP fluorescence signal was scattered in the epidermal cells of the *Nicotiana benthamiana* leaves (**Figure 3**). The results showed that the GmABCG5 protein was located at the cell plasma membrane.

### 3.4 *GmABCG5* expression responded to iron deficiency

**Figure 4:**
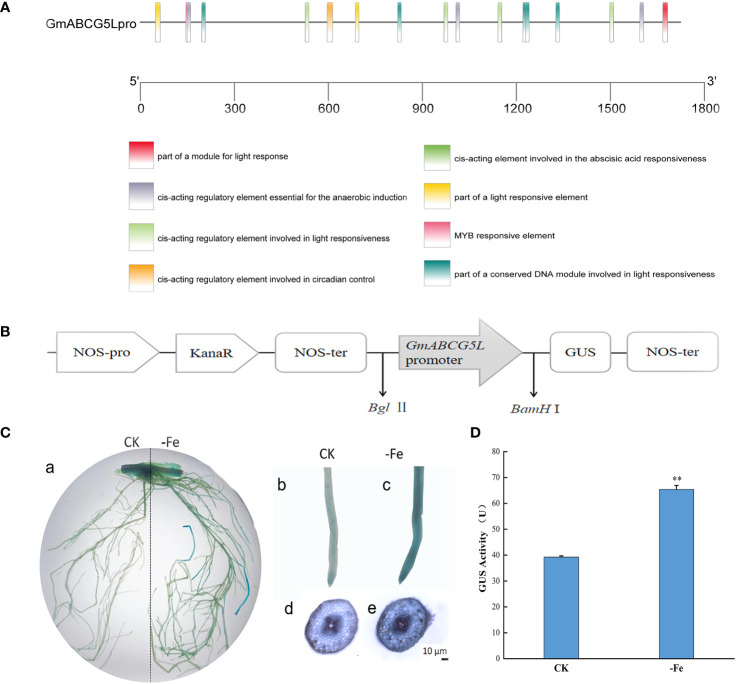
Response of the *GmABCG5* promoter and *GmABCG5* expression to iron deficiency. **(A)** Several predicted cis-elements in the *GmABCG5* promoter. **(B)** The pCAMBIA1301-*GmABCG5*pro::GUS recombinant expression vector. **(C)** GUS staining of *GmABCG5*pro::GUS-transformed hairy root of soybean under iron deficiency for 7 d, with non-stress treatment as the CK. (a) The hairy root of soybean. (b) Staining of a soybean hairy root tip under CK. (c) Staining of a soybean hairy root tip under iron deficiency stress. (d) Transverse cutting view of a hairy root under non-stress treatment. **(e)** Transverse cutting view of hairy root under iron deficiency treatment. Bar = 10 µm. **(D)** GUS enzyme activity of *GmABCG5*pro::GUS transformed soybean hairy root under iron deficiency for 7 d, with non-stress treatment as the CK. The experiments were repeated three times, and each treatment group contained five samples. The data represent the mean of the replicates. * indicates *p*<0.05, ** indicates *p*< 0.01.

In addition to the core cis-elements, the *GmABCG5* promoter (*GmABCG5*pro) contains a large number of light-responsive elements, MYB binding sites, and ABA response elements (**Figure 4A**). The expression vector pCAMBIA1301-*GmABCG5*pro::GUS was constructed (**Figure 4B**), and the hairy root was induced by Agrobacterium-mediated acupuncture infection on soybean. The hairy roots of soybean showed blue staining under non-stress and iron deficiency treatments. The staining was deeper in soybean hairy roots under iron deficiency than under non-stress (**Figure 4C**). The results showed that *GmABCG5*pro could be induced by iron deficiency. The GUS enzyme activity of transgenic *GmABCG5*pro::GUS soybean hairy root was determined. The results showed that the GUS enzyme activity in *GmABCG5*pro::GUS soybean hairy roots was significantly higher than that of the control under iron deficiency stress (*p*<0.05), which was consistent with the results of GUS histochemical staining (**Figure 4D**; **Supplementary Figure 1**). The qRT-PCR results showed that *GmABCG5* expression was significantly increased in stem and leaf under iron deficiency (*p*<0.05) compared with non-stress treatment (**Figure 2C**). The qRT-PCR result was consistent with the GUS assay, indicating that the *GmABCG5* gene expression was induced by iron deficiency in the roots, the stem, and the leaf of soybean, especially in the leaf.

### 3.5 Growth of *GmABCG5* in the soybean complex

**Figure 5:**
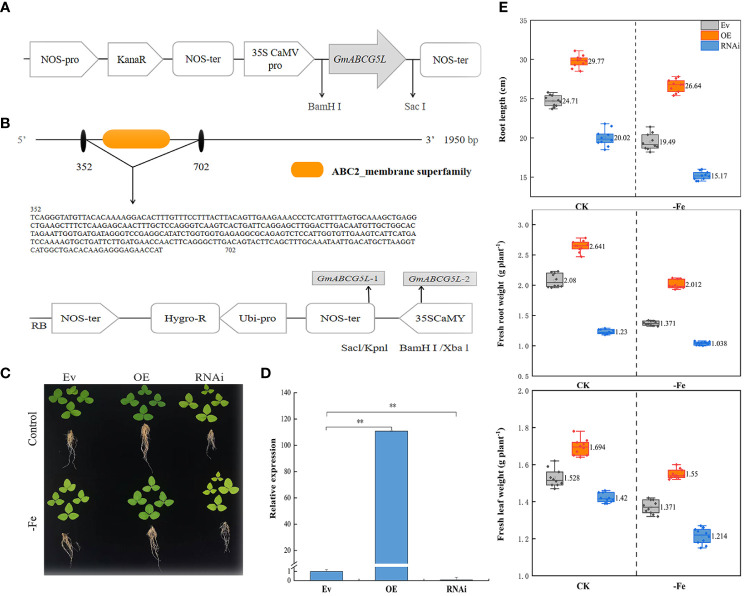
The phenotypes of OE*GmABCG5*, i*GmABCG5*, and Ev under iron deficiency. **(A)** The construction of the pROKII-*GmABCG5* recombinant vector. **(B)** Construction of the pZH01-*GmABCG5* recombinant vector. **(C)** The OE*GmABCG5*, i*GmABCG5*, and Ev were hydroponically cultivated in 1/2 Hoagland solution and treated with iron deficiency (0 mM Fe) for 7 d, the control was hydroponically grown in 1/2 Hoagland solution for equal time. **(D)** *GmABCG5* expression in the hairy roots of OE*GmABCG5*, i*GmABCG5*, and Ev. ** indicates *p <*0.01. **(E)** The root length, the fresh root weight, and the fresh leaf weight of OE*GmABCG5*, i*GmABCG5*, and Ev complexes.

The pROKII-*GmABCG5* was constructed and transformed into soybean. The 350-bp RNA interference target fragment (352-702) of the ABC2 membrane domain in the *GmABCG5* gene was identified, and the RNAi vector pZH01-*GmABCG5* was constructed (**Figures 5A, B**). Compared with Ev, OE*GmABCG5* displayed longer hairy roots and greener leaves, and i*GmABCG5* displayed shorter hairy roots and yellower leaves under iron deficiency (**Figure 5C**). The OE*GmABCG5*, i*GmABCG5*, and Ev soybean complexes were obtained by Agrobacterium-mediated acupuncture infection. The qRT-PCR results showed that *GmABCG5* expression was significantly increased in OE*GmABCG5* hairy roots and was significantly decreased in i*GmABCG5* hairy roots, compared with Ev (*p*<0.01) (**Figure 5D**).

The root length, fresh root weight, and fresh leaf weight of OE*GmABCG5*, i*GmABCG5*, and Ev in the control were all higher than those under iron deficiency. The results proved that iron deficiency has a negative effect on the growth of soybean. The root length, the fresh root weight, and the fresh leaf weight in OE*GmABCG5* were higher than those in Ev, while these indices in i*GmABCG5* were lower than those in Ev. The results indicated that overexpression of *GmABCG5* improved the plant growth of soybean complexes, while inhibition of *GmABCG5* was stunted (**Figure 5E**).

### 3.6 Chlorophyll content and FCR activity

**Figure 6:**
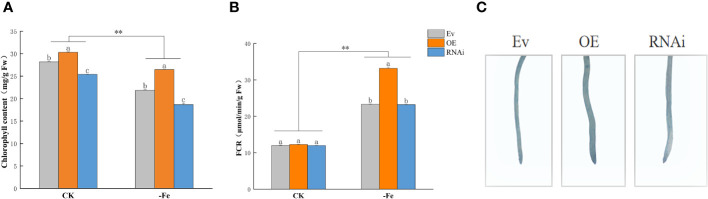
Chlorophyll content, FCR activity, and Perls staining in OE*GmABCG5*, i*GmABCG5*, and Ev. **(A)** Chlorophyll content. **(B)** FCR activity. **(C)** Perls staining. The OE*GmABCG5*, i*GmABCG5*, and Ev were grown in 1/2 Hoagland solution for 14 d and transferred to 1/2 Hoagland solution with the CK (0.1 mM Fe) or iron deficiency (0 mM Fe) for 7 d. Experiments were repeated three times, and each treatment group contained five samples. Data represent the mean of the replicates. ** indicates *p*<0.01, the statistical difference between the normal condition and iron deficiency treatment groups. Lowercase letters indicate *p*<0.05, the statistical difference between OE*GmABCG5*, i*GmABCG5*, and Ev in the same treatment group.

Chlorophyll content was significantly higher in OE*GmABCG5*, i*GmABCG5*, and Ev s than those under iron deficiency (*p*<0.01). Compared with Ev, the chlorophyll content was significantly higher in OE*GmABCG5* and was significantly lower in i*GmABCG5* under the control and iron deficiency treatment (*p*<0.05) (**Figure 6A**). Combining the phenotypic changes, the results indicated that overexpression of *GmABCG5* improved, while inhibition of *GmABCG5* stunted chlorophyll synthesis regardless of iron deficiency (**Figure 5C**). FCR activity was significantly higher in OE*GmABCG5*, i*GmABCG5*, and Ev under iron deficiency than that under the control (*p*<0.01). There was no significant difference in FCR activity among the three soybean complexes in the control. After iron deficiency treatment, the FCR activity of OE*GmABCG5* was significantly higher than that of i*GmABCG5* and Ev (*p*<0.01). The increase in FCR activity of OE*GmABCG5* caused by iron deficiency was significantly higher than that of i*GmABCG5*, and Ev (**Figure 6B**). The results indicated that when the plant was subjected to iron deficiency, overexpression of *GmABCG5* activated FCR activity, while inhibition of *GmABCG5* did not impact FCR activity. A Perls staining in the OE*GmABCG5* hairy root showed the deepest, and i*GmABCG5* showed the lightest color (**Figure 6C**). The results showed that OE*GmABCG5* had the most Fe accumulation and i*GmABCG5* had the least Fe accumulation.

### 3.7 Physiological indices

**Figure 7:**
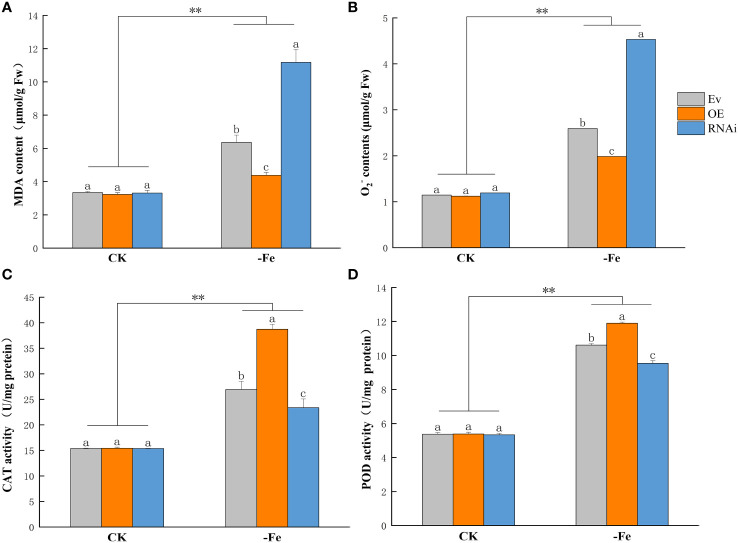
Physiological indices of OE*GmABCG5*, i*GmABCG5*, and Ev under iron deficiency. **(A)** Malondialdehyde content. **(B)** Superoxide anion content. **(C)** Catalase activity. **(D)** Peroxidase activity. The soybean complexes were grown in 1/2 Hoagland solution for 14 d and transferred to 1/2 Hoagland solution with the control (0.1 mM Fe) or iron deficiency (0 mM Fe) for 7 d. The experiments were repeated three times, and each treatment group contained five samples. Data represent the mean of the replicates. ** indicates *p*<0.01, the statistical difference between the normal condition and iron deficiency treatment groups. Lowercase letters indicate *p*<0.05, the statistical difference in OE*GmABCG5*, i*GmABCG5*, and Ev in the same treatment group.

Malondialdehyde (MDA) and Superoxide anion (O_2_^-^) contents were significantly higher in OE*GmABCG5*, i*GmABCG5*, and Ev under iron deficiency than in the control (*p*<0.01). MDA and O_2_^-^ contents showed no significant difference in OE*GmABCG5*, i*GmABCG5*, and Ev in the control. Under iron deficiency, = MDA and O_2_^-^ contents were significantly lower in OE*GmABCG5* and significantly higher in i*GmABCG5* than in Ev (*p*<0.05) (**Figures 7A, B**). The results indicated that overexpression of *GmABCG5* reduced oxidative damage, while inhibition of *GmABCG5* increased oxidative damage in plants under iron deficiency. In the control, the activities of peroxidase (POD) and catalase (CAT) showed no significant difference in OE*GmABCG5*, i*GmABCG5*, and Ev. After 7 d of iron deficiency, POD and CAT activities were significantly increased in the three soybean complexes compared with the control (*p*<0.01). The POD and CAT activities were significantly lower in OE*GmABCG5* and significantly higher in i*GmABCG5* than in Ev (*p*<0.05) (**Figures 7C, D**). The results indicated that overexpression of *GmABCG5* enhanced, while inhibition of *GmABCG5* reduced, the antioxidant capacity of plants under iron deficiency.

## 4 Discussion

Due to their ability to transport compounds in multiple biological processes, the applicability of ABC transporters in biotechnology is enormous. Evolutionary analysis showed that soybean GmABCG5 is closely related to AtABCG5 in Arabidopsis and OsABCG5 in *Oryza sativa*, with similar structures (**Figures 1A, B**), which indicates that *ABCG5* genes are evolutionarily conserved. The characterization of *GmABCG5* will also provide useful information about other function-unknown *GmABCG* genes.

We found that the expression of *GmABCG5* in the reproductive organs was higher than that in the vegetative organs, which is consistent with the previous finding that *GmABCG5* (Glyma.05G192700) was upregulated in the pod and seed of William82 soybean (Mishra et al., 2019). *GmABCG5* may play an essential role in soybean reproduction, seed development, and maturation. Previous research also showed that *ABCG5* expression is present at low levels in the roots of soybean (Mishra et al., 2019) and Arabidopsis (Lee et al., 2021), with no expression even in 6-week-old *Medicago truncatula* (Jasinski et al., 2009). On the contrary, in our study, the expression of *GmABCG5* was higher in roots than in leaves. We speculated that the difference in tissue expression of these *ABCG5* genes might be due to differences in species and functions. *GmABCG5* may play a role in soybean roots.

In this study, *GmABCG5* expression was significantly up-regulated in the roots and was dramatically up-regulated in the leaves of soybean under iron deficiency. GUS staining also showed that *GmABCG5*pro responded to iron deficiency (**Figure 4C**). Upregulation of iron transporter genes such as *YSL* (Senoura et al., 2017) and *IRT* (Huang and Dai, 2015) is an important strategy for plants to overcome iron deficiency because iron transporters could take up and redistribute iron and improve plant growth. Meanwhile, plants secrete organic substances that promote iron absorption. For example, members of the ABCG subfamily, *AtABCG37*, and *NtABCG3*, were strongly induced by iron deficiency in plant roots, secreting coumarins to promote iron absorption in the plant rhizosphere (Rodríguez-Celma et al., 2013; Fourcroy et al., 2014; Ziegler et al., 2017). The up-regulation of *GmABCG5* by iron deficiency confirms that *GmABCG5* is involved in the iron deficiency response, especially in the leaf. Our result is in contrast to the studies of Lefevre et al., which found that the *GmABCG5* gene in stems and leaves was not induced by iron deficiency. *GmABCG5* was located at the cell plasma membranes, which is consistent with the characteristics of transporters (Andolfo et al., 2015). When plants suffer from iron deficiency, iron transporters mobilize and redistribute Fe in the leaves to supply iron to organs that preferentially require Fe. Thus, we suggest that *GmABCG5* may have the function of mobilizing and redistributing Fe and/or secreting Fe substances to maintain iron homeostasis in plants.

We found that the *GmABCG5* promoter (*GmABCG5*pro) contains a large number of photoresponsive elements, MYB binding sites, and abscisic acid response elements, suggesting a role for *GmABCG5*’ in development and abiotic stress response (Mishra et al., 2019). The existence of several ABA-responsive elements in *GmABCG5*pro suggests that *GmABCG5* could be induced by ABA. *OsABCG5* has been shown to be associated with ABA in guard cells. Several reports have linked ABA to heavy metal tolerance responses, such as lead-related phenotypes, which are indirect effects of the ABA transport function of *AtPDR12* (Atici et al., 2005; Fan et al., 2014). The relationship between auxin and *ABCG5* has been discussed in previous works of literature. Although some ABCGs have been observed to be involved in the auxin pathway (Strader and Bartel, 2009), *GmABCG5* is unlikely to be related to auxin as there is no auxin-responsive element in its promoter. *GmABCG5* may directly affect root growth and development independently of auxin signaling, like *OsABCG5*, its homologous gene in rice. In this study, overexpressed *GmABCG5* affects hairy root elongation, whether under iron deficiency treatment or not.

In this study, soybean hairy root growth is promoted by *GmABCG5* and retarded by *GmABCG5* inhibition, which further confirms the previously discovered growth-promoting function of *ABCG5*. The worse growth status of the soybean complex seedlings under iron deficiency confirms the importance of iron nutrients for plant growth (Yasuno et al., 2009). Iron deficiency has a very obvious inhibitory effect on hairy root elongation in soybean. At the same time, we also see that the contribution of *GmABCG5* to hairy root elongation is closely related to iron nutrition. The rate of root length reduction caused by iron deficiency was the lowest in OE*GmABCG5* and the highest in i*GmABCG5*. The result means that *GmABCG5* could save the delayed hairy root elongation phenotype and that the knockdown of *GmABCG5* has a synergistic effect with iron deficiency to delay hairy root elongation. The transport function of the transporter is conducive to substance synthesis and biological growth (Briat et al., 2015; Prity et al., 2021; Barnaby et al., 2022), which may explain the high biomass of OE*GmABCG5*. Here, the decrease in fresh weight of Ev, OE*GmABCG5*, and i*GmABCG5* caused by iron deficiency may be due to the inhibition of iron-participating biological processes. It is very interesting that the decrease in plant root biomass caused by iron deficiency was alleviated in i*GmABCG5*. This may be a compensatory effect of iron deficiency, but the exact reason is not clear.

Chlorophyll content and FCR activity are both valuable parameters for the state of plant iron nutrition. Overexpression of *GmABCG5* increased chlorophyll content, and inhibition of *GmABCG5* decreased chlorophyll content, corresponding to the yellowed leaf phenotype. The higher chlorophyll content showed that the effect of iron deficiency on plants was reduced, indicating that *GmABCG5* can increase a plant’s tolerance to iron deficiency (Wakabayashi et al., 2006; Frelet-Barrand et al., 2008; Mirzahossini et al., 2015; He et al., 2020). The increase in FCR activity is a typical response to iron deficiency because FCR promotes iron absorption in plants. The increase in FCR activity induced by iron deficiency was much higher in OE*GmABCG5* than in the other two complexes. This result indicates that the response mechanism to iron deficiency in plants is more actively mobilized in OE*GmABCG5*, suggesting that *GmABCG5* is related to the iron deficiency response. Accordingly, the iron content is high in OE*GmABCG5* and low in i*GmABCG5*, which is consistent with previous studies (Sisó-Terraza et al., 2016; Dahuja et al., 2021). We also used Perls staining to visually demonstrate the iron content in plants, and the results showed that *GmABCG5* overexpressed plants had high iron content. The results suggest that the *GmABCG5* gene allows more iron to enter the plant, although the effects of other transporters cannot be excluded. Thus, we conclude that *GmABCG5* may be involved in iron absorption and regulate iron homeostasis in plants.

Iron and ROS have attracted much interest because of their sophisticated (Reyt et al., 2015; Le et al., 2016; vonder Mark et al., 2021; Hornbergs et al., 2023). Furthermore, no difference was found in the three soybean complexes in the control, while under iron deficiency, less membrane damage and superoxide anion and higher antioxidant capacity were found in OE*GmABCG5*, and the opposite result was found in i*GmABCG5*. This difference indicates that *GmABCG5* could counteract the damage caused by iron deficiency and improve the ability to protect against oxidative damage, which suggests a close relationship between *GmABCG5* and iron (Dhara and Raichaudhuri, 2021). In plant tissues, severe iron deficiency stress catalyzes the production of reactive oxygen species (ROS), which cause irreversible damage to membrane lipids, proteins, and nucleic acids (Becana et al., 1998; Zingg and Jones, 1997). MDA, an indicator of oxidative stress, was found to be increased by iron deficiency stress in alfalfa (M’Sehli et al., 2014), similar to our results that MDA content increased significantly in soybean after iron deficiency. We also found that the MDA content increased less when GmABCG5L was overexpressed, indicating that oxidative damage was reduced in plants. Under iron deficiency, plants can reduce or remove the active oxygen in the cell to avoid the harm caused by the accumulation of ROS through the synergistic action of the SOD, CAT, and POD. It was found that iron deficiency inhibited soybean growth, plant growth slowed down, and the activities of antioxidant enzymes CAT and POD showed an increasing trend (Mahmoud et al., 2022), which is consistent with our results. The significantly higher antioxidant enzyme activity in OE*GmABCG5* and lower in i*GmABCG5* suggested that overexpression of *GmABCG5L* can produce more protective enzymes, effectively remove harmful substances, and resist oxidative damage induced by iron deficiency. However, Wang et al. found that iron deficiency culture had no obvious effect on the CAT activity of tobacco leaves but reduced their POD activity (Thoiron et al., 1997). The results of this study were inconsistent with theirs, which may be due to the inconsistent response of different species to iron deficiency.

In conclusion, the soybean *GmABCG5* gene was cloned and characterized in this study. As an ABCG family transporter, soybean GmABCG5 is localized at the cell membrane. The *GmABCG5* promoter (*GmABCG5*pro) contains a large number of stress response elements. The expression of GmABCG5 was high in the reproductive organs of soybean plants. Soybean *GmABCG5* gene expression was induced by iron deficiency, especially in the leaf. Overexpression/inhibition of *GmABCG5* in soybean hairy roots proved that *GmABCG5* has a positive effect on plant growth. The results indicated that overexpression of *GmABCG5* increased, while inhibition of *GmABCG5* reduced, the tolerance capacity to iron deficiency in plants. The results elucidate the function of the *GmABCG5* gene and provide new insights into the regulation of iron homeostasis in soybean.

## Data availability statement

The original contributions presented in the study are publicly available. This data can be found here: https://www.ncbi.nlm.nih.gov/gene/?term=LOC100789722.

## Author contributions

YW: Writing – original draft. XZ: Writing – original draft. YY: Writing – original draft. TN: Writing – original draft. MZ: Writing – review & editing, Conceptualization, Project administration. CF: Writing – original draft. WL: Writing – original draft. YS: Writing – original draft. CG: Writing – review & editing. DG: Writing – original draft. YB: Writing – review & editing.
