# Supplementary material for: GmABCG5, an ATP-binding cassette G transporter gene, is involved in the iron deficiency response in soybean
Source: Front Plant Sci. 2024 Jan 5;14:1289801. doi: 10.3389/fpls.2023.1289801 (PMC10796643; doi:10.3389/fpls.2023.1289801)
Supplement: Supplementary file 1 [file DataSheet_1.pdf]

Supplementary Material

Supplementary Table 1 The Primers used in this experiment

Primer	Base sequence（5’-3’）	Purpose
GmABCG5 S	CGGGATCCATGATGCAAGGCTGTGAG	Cloning of
GmABCG5 A	CGAGCTCTTAGTTGGTGACAACCCTG	*GmABCG5*
GmABCG5 S	GGGTACAGAAACAACATC	
GmABCG5 A	AATAATCCCAACAGTGAG	Quantitative RT-
GmACTIN S	ACGAGCGTTTCAGATG	PCR
GmACTIN A	ACCTCCGATCCAGACA	
*GmABCG5*pro S	CGGGATCC TCATAGATTAGGCTTTCACC	Cloning of
*GmABCG5*pro A	GAAGATCT GGTTTGAATAGGTTGGCT	*GmABCG5* promoter
GmABCG5 S	CCAAGCTTTATCTGAGTCATGCCATAC	subcellular
GmABCG5 A	CGGGATCCGAAAAAAAGGGTGAACG	localization
GmABCG5-1 S	CGGGATCCTCAGGGTATGTTACACAAAAGG	
GmABCG5-1 *A*	GCTCTAGAATGGTTCTCCCTCTTGTGTCA	
GmABCG5-2 S	CGAGCTCTCAGGGTATGTTACACAAAAGG	RNAi
GmABCG5-2 A	GGGTACCATGGTTCTCCCTCTTGTGTCA	

Supplementary Table 2 The 67 known ABCG transporters in Arabidopsis thaliana, Glycine max and Oryza sativa.

Name	Plant species	Accession
AtABCG9	*Arabidopsis thaliana*	NP_194472.3
AtABCG21	*Arabidopsis thaliana*	NP_001189973.1
AtABCG28	*Arabidopsis thaliana*	NP_200882.4
AtABCG25	*Arabidopsis thaliana*	NP_565030.1
AtABCG40	*Arabidopsis thaliana*	NP_173005.1
AtABCG37	*Arabidopsis thaliana*	NP_190916.1
AtABCG29	*Arabidopsis thaliana*	NP_566543.1
AtABCG30	*Arabidopsis thaliana*	NP_001319944.1
AtABCG14	*Arabidopsis thaliana*	NP_564383.1
AtABCG39	*Arabidopsis thaliana*	NP_176867.2
AtABCG41	*Arabidopsis thaliana*	NP_001329359.1
AtABCG32	*Arabidopsis thaliana*	NP_180259.1
AtABCG33	*Arabidopsis thaliana*	NP_001325068.1
AtABCG35	*Arabidopsis thaliana*	NP_172973.1
AtABCG34	*Arabidopsis thaliana*	NP_181179.2
AtABCG27	*Arabidopsis thaliana*	NP_001319729.1
AtABCG26	*Arabidopsis thaliana*	NP_187927.1
AtABCG31	*Arabidopsis thaliana*	NP_180555.2
AtABCG5	*Arabidopsis thaliana*	NP_178984.1
AtABCG22	*Arabidopsis thaliana*	NP_568169.1
AtABCG7	*Arabidopsis thaliana*	NP_849921.1
AtABCG42	*Arabidopsis thaliana*	NP_001190737.1
OsABCG5L	*Oryza sativa*	NP_001404431.1
OsABCG37L	*Oryza sativa*	NP_001396179.1
OsABCG22	*Oryza sativa*	XP_015632516.1
OsABCG50L	*Oryza sativa*	XP_015618070.1
OsABCG28	*Oryza sativa*	XP_025877343.1
OsABCG52L	*Oryza sativa*	XP_015610794.1
OsABCG49L	*Oryza sativa*	XP_015620170.1
OsABCG46L	*Oryza sativa*	XP_015611135.1
OsABCG25L	*Oryza sativa*	NP_001409972.1
OsABCG3	*Oryza sativa*	XP_015627864.1
OsABCG11	*Oryza sativa*	XP_015611080.1
OsABCG40	*Oryza sativa*	NP_001403507.1
OsABCG41L	*Oryza sativa*	XP_015626628.1
GmABCG6	*Glycine max*	XP_003556547.1
GmABCG39	*Glycine max*	XP_006604625.1
GmABCG25	*Glycine max*	XP_003555426.1
GmABCG24	*Glycine max*	XP_003518985.1
GmABCG22	*Glycine max*	XP_006575266.1
GmABCG20	*Glycine max*	XP_003520635.1
GmABCGSTR	*Glycine max*	XP_003525183.1
GmABCG36	*Glycine max*	XP_003530098.1
GmABCG23	*Glycine max*	XP_003529479.3
GmABCG5	*Glycine max*	XP_003525127.1
GmABCG2	*Glycine max*	XP_025985323.1
GmABCG11	*Glycine max*	XP_003534067.1

GmABCG7	*Glycine max*	XP_003536632.1
GmABCG15	*Glycine max*	XP_003537732.1
GmABCG28	*Glycine max*	XP_014619646.1
GmABCG3	*Glycine max*	XP_003540196.1
GmABCG1	*Glycine max*	XP_003543921.2
GmABCG9	*Glycine max*	XP_003541547.2
GmABCG10	*Glycine max*	XP_003543218.2
GmABCG26	*Glycine max*	XP_003544606.1
GmABCG31	*Glycine max*	XP_003544291.1
GmABCG14	*Glycine max*	XP_003547739.1
GmABCG32	*Glycine max*	XP_003549791.1
GmABCG15L	*Glycine max*	XP_040869752.1
GmABCG32L	*Glycine max*	XP_040868797.1
GmABCG14L	*Glycine max*	XP_040873470.1
GmABCG21L	*Glycine max*	XP_040861356.1
GmABCG31L	*Glycine max*	XP_014619080.1
GmABCG11L	*Glycine max*	NP_001352052.1
GmABCG36L	*Glycine max*	XP_040873673.1
GmABCG39L	*Glycine max*	XP_040870073.1
GmABCG40L	*Glycine max*	XP_040860791.1

Supplementary Table 3 Chromosome distribution of *GmABCG* superfamily genes in soybean genome.

Gene name	Gene ID	Chromosome	Chromosome	Gene numbers
GmABCG26	Glyma.03G204800	Gm03	Gm03	5
GmABCG15L	Glyma.03G135525	Gm03	Gm05	2
GmABCG20	Glyma.03G175300	Gm03	Gm06	1
GmABCG39L	Glyma.03G192700	Gm03	Gm07	3
GmABCG22	Glyma.03G204800	Gm03	Gm08	4
GmABCG32	Glyma.05G011900	Gm05	Gm09	3
GmABCG5	Glyma.05G192700	Gm05	Gm10	4
GmABCG9	Glyma.06G248800	Gm06	Gm11	4
GmABCG9L	LOC100808846	Gm07	Gm12	2
GmABCG14L	LOC121175201	Gm07	Gm13	2
GmABCG23	Glyma.07G231400	Gm07	Gm14	1
GmABCG11L	Glyma.08G070800	Gm08	Gm15	1
GmABCG1L	LOC100775718	Gm08	Gm19	2
GmABCG2	Glyma.08G071100	Gm08	Gm20	6
GmABCG26L	Glyma.08G332100	Gm08	Total	40
GmABCG21L	Glyma.09G074732	Gm09		
GmABCG40L	Glyma.09G130700	Gm09		
GmABCG11	Glyma.09G160000	Gm09		
GmABCG12L	Glyma.10G113802	Gm10		
GmABCG24	Glyma.10G209200	Gm10		
GmABCG8L	LOC100805972	Gm10		
GmABCG7	Glyma.10G264900	Gm10		
GmABCG14	Glyma.11G090200	Gm11		
GmABCG15	Glyma.11G093800	Gm11		
GmABCG28	Glyma.11G196100	Gm11		
GmABCG31L	LOC106795059	Gm11		
GmABCG3	Glyma.12G177200	Gm12		
GmABCG10	Glyma.12G228500	Gm12		
GmABCG1	Glyma.13G044000	Gm13		
GmABCG36	Glyma.13G355000	Gm13		
GmABCG31	Glyma.14G193300	Gm14		
GmABCG35L	LOC100789938	Gm15		
GmABCG36L	Glyma.19G192900	Gm19		
GmABCG39	Glyma.19G192900	Gm19		
GmABCG3L	Glyma.20G052300	Gm20		
GmABCG7L	LOC100804521	Gm20		
GmABCG8L	LOC100805972	Gm20		
GmABCG25	Glyma.20G174800	Gm20		
GmABCG21	Glyma.20G184900	Gm20		
GmABCG6	Glyma.20G242000	Gm20		

Supplementary Table 4. The individual *P* value of physiological parameters in Figure 5-7 by one way ANOVA , Tukeys HSD.

Index / P value / Genotype Pairs		Root length	Fresh weight of root	Fresh weight of leaf	Chlorophyll	FCR	MDA	O_2^-	CAT	POD
CK	Ev/OE	0.005	0.001	0.000	0.000	0.941	0.505	0.992	0.931	0.992
	Ev/RNAi	0.008	0.048	0.000	0.000	0.933	0.942	0.967	0.997	0.818
	OE/RNAi	0.000	0.000	0.000	0.000	0.974	0.688	0.927	0.903	0.878
-Fe	Ev/OE	0.001	0.002	0.000	0.000	0.000	0.009	0.028	0.000	0.000
	Ev/RNAi	0.018	0.014	0.001	0.000	0.888	0.000	0.000	0.013	0.000
	OE/RNAi	0.000	0.000	0.000	0.000	0.000	0.000	0.000	0.000	0.000

Supplementary Table 5. The df and F statistic of physiological parameters in Figure 5-7 by one way ANOVA , Tukeys HSD.

Index / Genotype Pairs		Root length	Fresh weight of root	Fresh weight of leaf	Chlorophyll	FCR	MDA	O_2^-	CAT	POD
CK	df1	2	2	2	2	2	2	2	2	2
	df2	6	6	6	6	6	6	6	6	6
	df	8	8	8	8	8	8	8	8	8
	F	49.113	59.609	276.692	3051.625	0.058	0.744	0.072	0.108	0.211
	P	0.000	0.000	0.000	0.000	0.944	0.000	0.000	0.000	0.000
-Fe	df1	2	2	2	2	2	2	2	2	2
	df2	6	6	6	6	6	6	6	6	6
	df	8	8	8	8	8	8	8	8	8
	F	56.478	52.977	205.519	1286.879	1919.28	132.656	121.537	125.171	351.070
	P	0.000	0.000	0.000	0.000	0.000	0.000	0.000	0.000	0.000

Note: df1: Degree of freedom between groups. df2: Degree of freedom within a group. df(degree of freedom) = df1+df2.

Supplementary Table 6. The individual P value, df and F statistic of GmABCG5 gene expression in Figure 2 and Figure 5 and GUS activity in Figure 4 by one way ANOVA , Tukeys HSD test.

Genotype Pairs		P value	df1	df2	df	F
CK	Root/Stem	0.777	2	6	8	11.636
	Root/Leaf	0.007				
	Stem/Leaf	0.005				
	Flower/Pot	0.062	2	6	8	10.787
	Flower/Seed	0.009				
	Pot/Seed	0.282				
-Fe	Root	0.000	1	4	5	3108.947
	Stem	0.007	1	4	5	26.742
	Leaf	0.000	1	4	5	1699764.698
Hairy root of soybean	Ev	0.000	2	6	8	1294.447
	OE	0.161				
	RNAi	0.000				
GUS activity		0.000	1	4	5	803.204

Note: df1: Degree of freedom between groups. df2: Degree of freedom within a group. df(degree of freedom) = df1+df2.

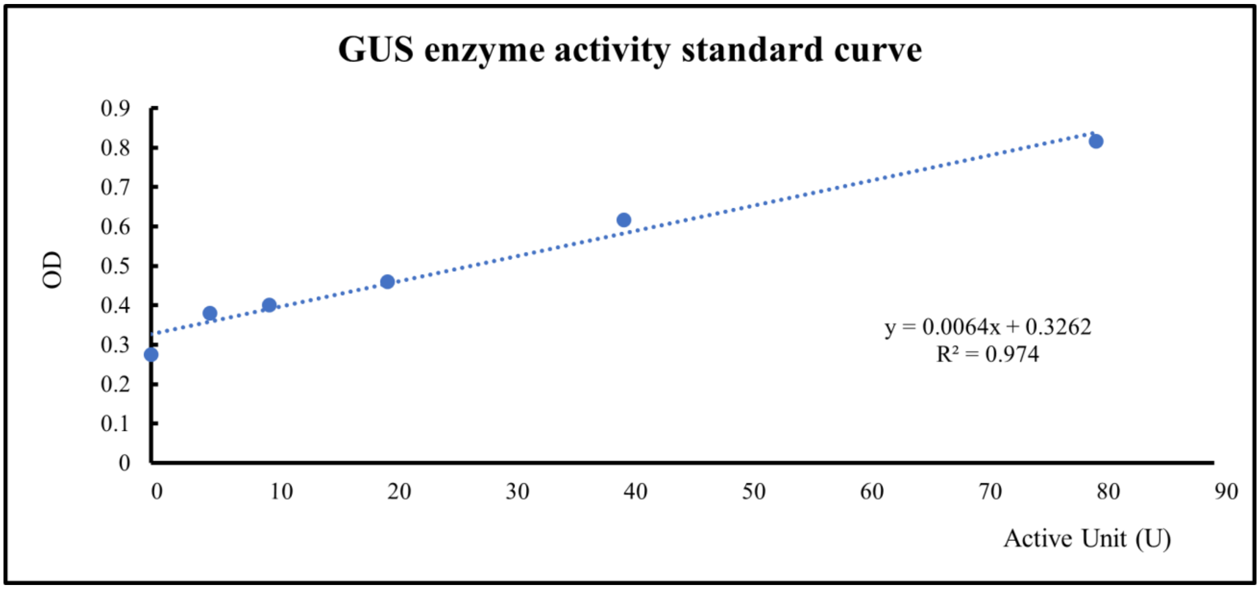

Supplementary Figure 1. GUS enzyme activity standard curve
